# *QuickStats:* Age-Adjusted Death Rates* for Motor Vehicle Traffic Injury^^† ^^— United States, 2016

**DOI:** 10.15585/mmwr.mm6731a4

**Published:** 2018-08-10

**Figure:**
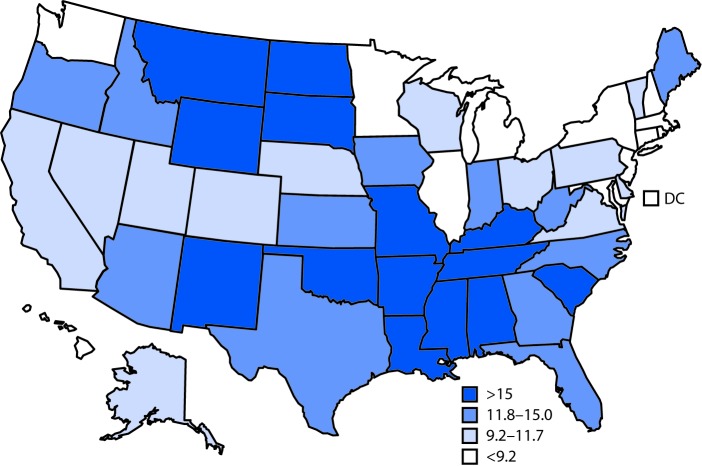
In 2016, the death rate in the United States for motor vehicle traffic injury was 11.7 per 100,000 standard population. The three states with the highest age-adjusted death rates were Mississippi (25.4), Alabama (23.3), and South Carolina (20.9). New York (5.3), Rhode Island (5.0), and the District of Columbia (4.5) had the lowest rates.

**Source: **National Vital Statistics System. Underlying cause of death data, 2016. https://wonder.cdc.gov/ucd-icd10.html.

For more information on this topic, CDC recommends the following link: https://www.cdc.gov/motorvehiclesafety/states/index.html.

